# Unusual Presentation of Prostate Cancer Metastatic to the Cricoid Cartilage and Oral Cavity

**DOI:** 10.1155/2018/5207204

**Published:** 2018-03-06

**Authors:** Marlene Olvera, Miriam Delgado, Melchor Vázquez, José Zavala, Verónica Macedo, Martha Puentes

**Affiliations:** ^1^Department of Radiotherapy, Centro Estatal de Oncología, San Francisco de Campeche, CAM, Mexico; ^2^Department of Pathology, Centro Estatal de Oncología, San Francisco de Campeche, CAM, Mexico; ^3^Department of Radiology, Centro Estatal de Oncología, San Francisco de Campeche, CAM, Mexico

## Abstract

In Mexico, prostate cancer is the second leading cause of death in men. Prostate cancer usually presents metastasis to the regional lymph nodes and bone. Hereby, we present an unusual case of metastatic prostate cancer, with affectation to the cricoid cartilage and oral cavity, being the first case to have ever been reported in Mexico. A 68-year-old Mexican man was diagnosed with prostate cancer and cribriform architecture histology with low serum level of prostate-specific antigen, debuting with laryngeal stridor. The biopsy came back positive for metastatic prostate carcinoma. During treatment with radiotherapy, metastasis developed to the oral cavity.

## 1. Introduction

Worldwide, prostate cancer is the second leading cause of death in men, with a peak incidence between 60 and 70 years of age. In Mexico, it is the second most frequent neoplasm and the second cause of death in men [1, 2]. Most patients with metastatic prostate cancer have high levels of prostate-specific antigen; there is, however, a subgroup (1%) of the population, which have either low or undetectable levels, so the use of androgen blockade is ineffective. Consequently, the prognosis of patients is quite poor [3]. Prostate cancer metastases often occur at sites such as the skeletal bone, lung, liver, pleura, and adrenal glands. In the literature, there are 14 published cases of metastasis to the larynx due to prostate cancer, which is a rare event in the clinical practice [4]. Metastases to the oral cavity are uncommon.

## 2. Case Presentation

A 68-year-old Mexican male with diabetes mellitus type 2 and systemic arterial hypertension was diagnosed in 2013 with prostate cancer by transurethral resection of the prostate which reported ductal adenocarcinoma not differentiated (Gleason score 9 (4 + 5)) and a prostate-specific antigen of 0.446 ng/dl. Initial treatment consisted of bilateral orchiectomy.

The same year, he attended our radiotherapy department, where further studies were performed: a computed tomography scan (CT scan) showed no tumor activity data and bone scintigraphy did not show any alterations with an increase to the tracer and cystoscopy which gave no indication of bladder infiltration. A clinical stage of high risk was declared for the patient.

Radical radiotherapy with a dose of 75 Gy in 42 sessions was recommended, with his treatment ending in July 2014. During the patient's surveillance, nadir of the prostate-specific antigen of 0.053 ng/dl was corroborated.

**Figure 1:**
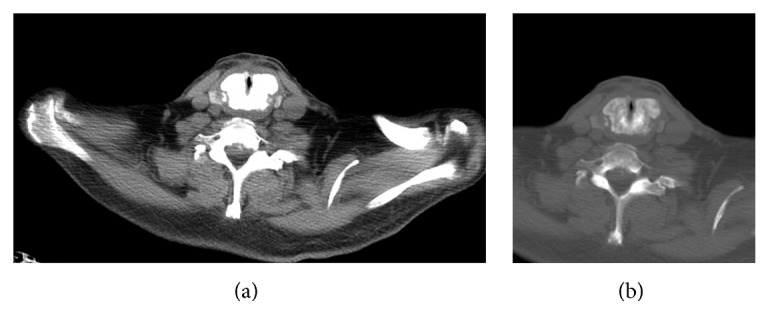
CT scan with axial sections showing enlargement and generalized sclerosis of the cricoid cartilage. Bone window where cricoid blast transformation becomes more evident.

In September 2016, the patient presented symptoms of dysphonia and laryngeal stridor. A CT scan was performed which shows decreased tracheal lumen and thickening of the thyroid and cricoid cartilages (Figure 1).

**Figure 2:**
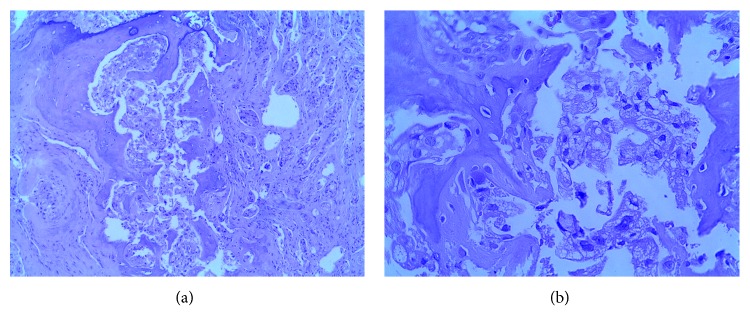
A cartilage is identified with destruction by infiltration of a glandular neoplasia, with formation of small nodules with microlens as well as individually. In other fields, it presents a cribriform pattern, where the presence of large and multiple nucleoli stands out. The neoplastic cells are of medium size, polygonal, with broad vacuolated cytoplasm, with large, hyperchromatic, and pleomorphic nuclei, and with a prominent nucleus and eosinophil.

A fibrolaryngoscopy revealed findings of extrinsic compression of the trachea from the membranous portion. An emergency tracheostomy was performed due to impending asphyxia, during which biopsies were taken. Metastatic carcinoma with the ductal cribriform pattern was corroborated (Figure 2). A new bone scintigraphy was requested, reporting disseminated disease positive in axial and appendicular to the larynx.

**Figure 3:**
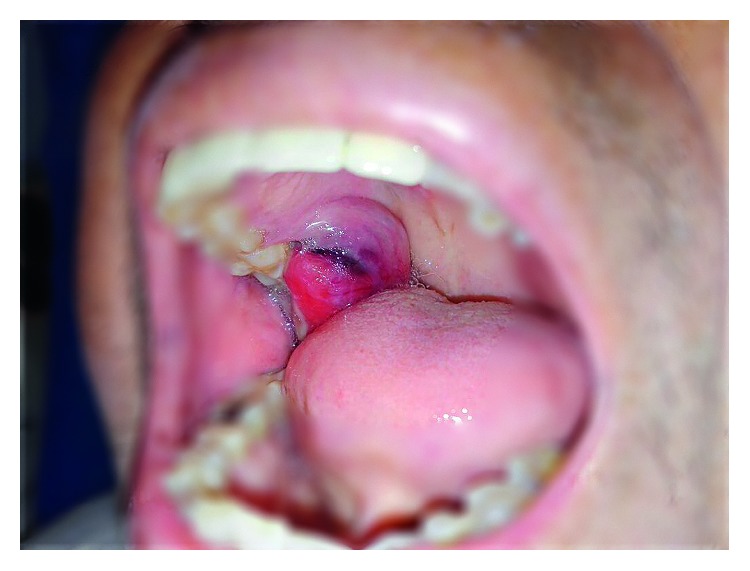
Oral cavity with a tumor of 2 cm in the right upper gingiva near to the third molar with extension to the hard palate.

**Figure 4:**
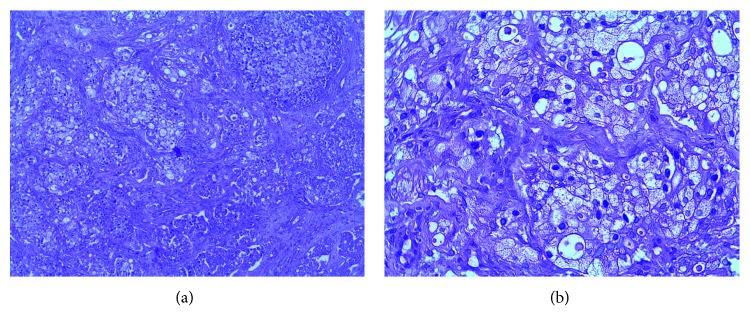
A neoplasm with an infiltration pattern is identified in small- and medium-sized nodules, surrounded by desmoplastic stroma. In some areas, minimal formation of tubular structures with low eosinophilic luminal secretion is identified. The cells that make up the neoplasia are large in size, with abundant cytoplasm, with a clear “foamy” aspect, with peripheral nuclei, and with some central, hyperchromatic, large, and regular borders.

For the previous reason, palliative radiotherapy was decided as treatment to the larynx, during which the patient presented a nodule in the oral cavity in the upper right gingival area with a hard palate extension (Figure 3). The biopsy reported a carcinoma with cribriform xanthomatous phenotype (Figure 4). The patient is currently receiving chemotherapy treatment based on docetaxel.

## 3. Discussion

Laryngeal metastasis is a rare entity, since the most frequent metastases to this organ are melanoma and renal carcinoma. The most affected anatomic site of the laryngeal is the supraglottis, whereas the glottis is the least common. The dissemination pathways might be hematogenous or lymphatic [5]. There have been 14 published cases since 1908 of this type of metastasis [4]. Laryngeal metastases usually develop unnoticed and may or may not present clinical symptoms, such as hoarseness, stridor, or develop infiltration into the supraglottic space with decrease in the tracheal lumen. The diagnosis is made by a CT scan, which plays an important role in the identification of the lesion and its extension. A nasolaryngoscopy biopsy is used to determine the primary origin [1, 5].

Metastases in the oral region are infrequent with only 1 to 1.5% prevalence, and there have been anecdotal findings reported in the literature, most frequently in the jaw and maxillary and in less proportion in the gingiva [4, 5, 7].

The case presented confirms what has been previously stated by the literature: it is a nonfavorable histology, a non-antigen-raising, and a non-symptom presenting during his surveillance. The use of the positron emission tomography-computed tomography (PET-CT) during the surveillance of patients with the same mentioned characteristics is of high importance for the early detection of metastases. Castration either surgical or biochemical may not have favorable results due to the aggressive behavior of the histology [8].

## 4. Conclusion

A case of metastatic carcinoma in the cricoid cartilage and oral cavity is reported, which can be considered as case number 15 in the clinical literature and the first reported case in Mexico. The present case aims to enrich the literature on metastatic cancer to infrequent sites in non-antigen-enhancer prostate cancer.

## References

[1] Elabbady A., Kotb A. F. (2013). Unusual presentations of prostate cancer: a review and case reports. *Arab Journal of Urology*.

[2] Gomez-Guerra L., Martinez-Fierro M., Alcantara-Aragon V. (2009). Population based prostate cancer screening in north Mexico reveals a high prevalence of aggressive tumors in detected cases. *BMC Cancer*.

[3] Lee D., Park J., Kim J. (2010). Progression of prostate cancer despite an extremely low serum level of prostate-specific antigen. *Korean Journal of Urology*.

[4] Prescher A., Schick B., Stütz A., Brors D. (2002). Laryngeal prostatic cancer metastases: an underestimated route of metastases?. *The Laryngoscope*.

[5] Oliveira J., Said R., Cartaxo R., Santos J., Gondim R. (2012). Metástase laríngea por adenocarcinoma de próstata: uma entidade rara. *Brazilian Journal of Otorhinolaryngology*.

[6] Abemayor E., Cochran A. J., Calcaterra T. C. (1983). Metastatic cancer to the larynx: diagnosis and management. *Cancer*.

[7] Manjunatha B., Kumar G. (2013). Metastatic tumors to the jaws and oral cavity. *Journal of Oral and Maxillofacial Pathology*.

[8] Satheesan B., Damodaran D., Kathiresan N. (2008). Oral cavity metastasis: an unusual presentation of carcinoma prostate. *Indian Journal of Urology*.

